# Supplementary figures and images for: Conserved domains and structural motifs that differentiate closely related Rex1 and Rex3 DEDDh exoribonucleases are required for their function in yeast
Source: PLoS One. 2025 Jun 2;20(6):e0321120. doi: 10.1371/journal.pone.0321120 (PMC12129344; doi:10.1371/journal.pone.0321120)

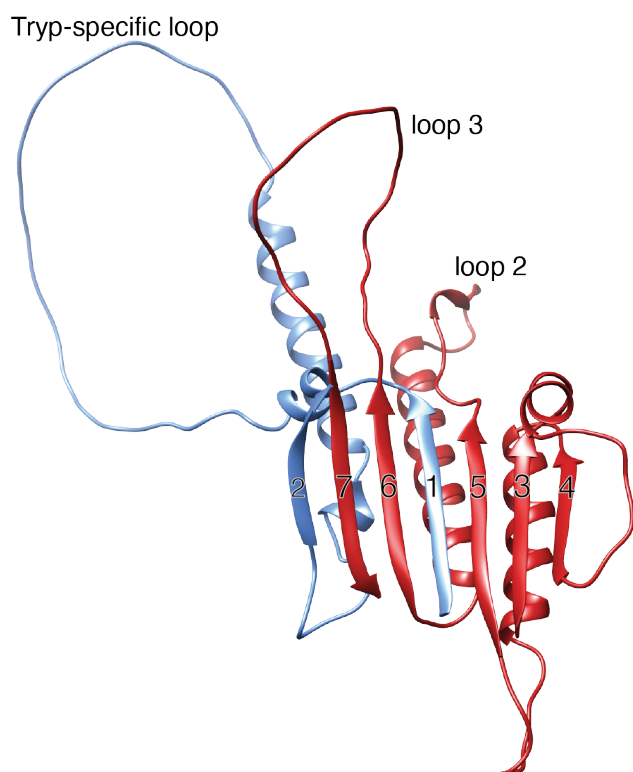

*T. brucei brucei* Rex1 AlkP domain

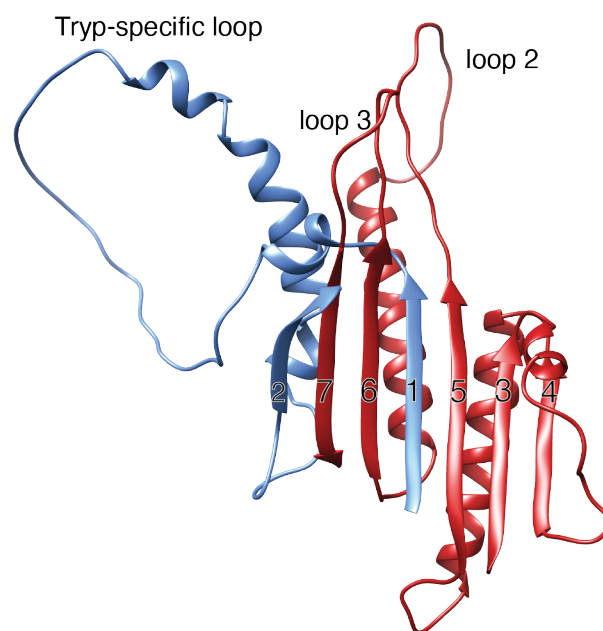

*T. cruzi* Rex1 AlkP domain

Supplement: S2 Figure — Ribbon structures of the AF models of the AlkP domains of Rex1 homologues from T. brucei brucei (AF-Q580Z1) and T. cruzi (AF-Q4DG67). The perspective shown is towards the surface of the ß-sheet, as shown for proteins in Figure 1. N-terminal sequences are coloured in blue. C-terminal sequences are coloured in red. For clarity, only sequences from strand 1 to strand 2 and from strand 3 to strand 7 are shown. (PDF) [file pone.0321120.s002.pdf]

Tree scale: 0.1

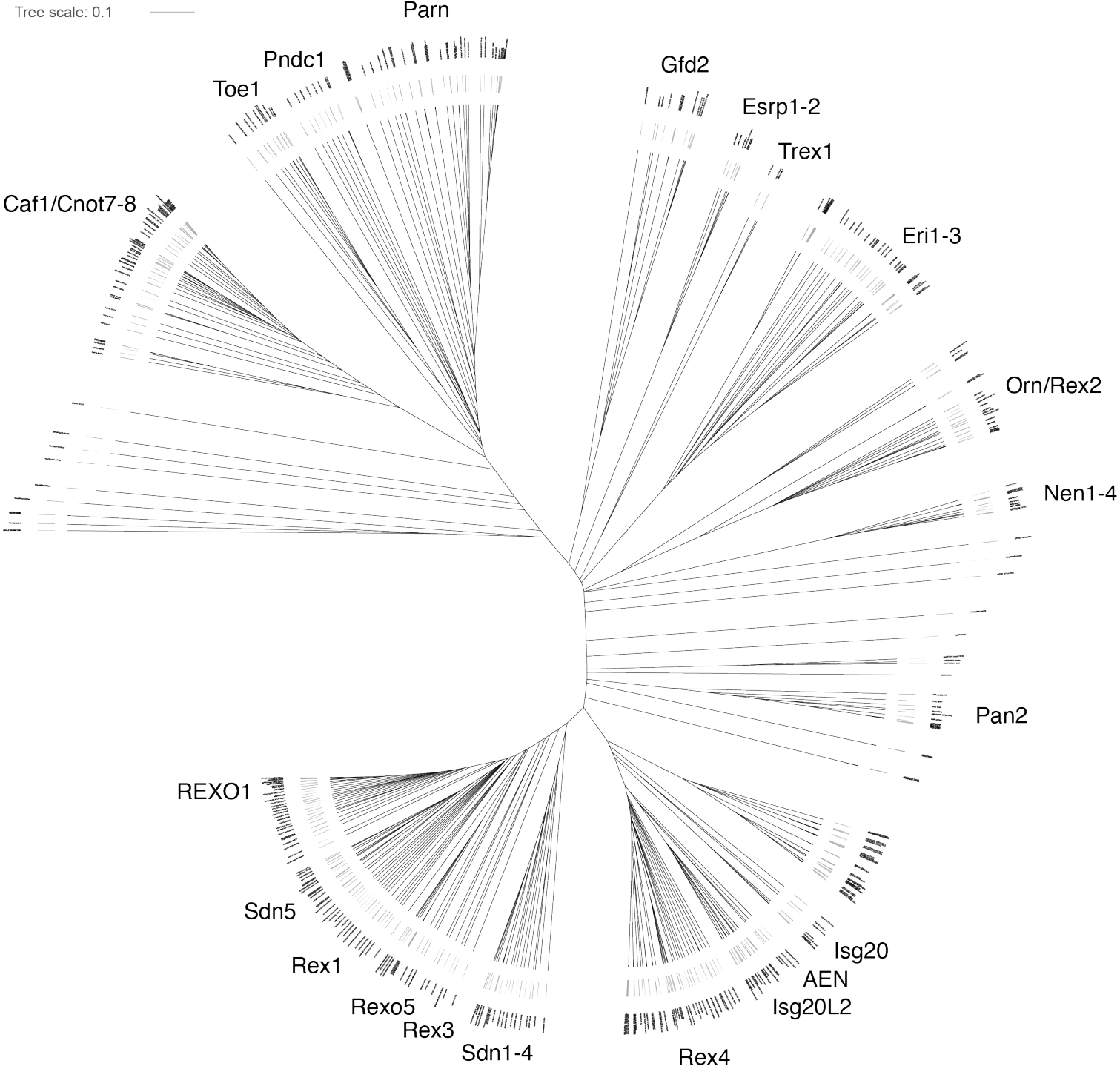

Supplement: S5 Figure — Unrooted phylogenetic tree of 387 distinct DEDDh domain sequences from diverse eukaryotic organisms. Proteins are labelled and annotated according to UniProt entries. Proteins from A. thaliana, C. elegans, S. cerevisiae, C. neoformans, C. reinhardtii, D. discoidium, D. rerio, D. melanogaster, chick and humans are indicated in bold type. The positions of major proteins within the tree are indicated. (PDF) [file pone.0321120.s005.pdf]

A

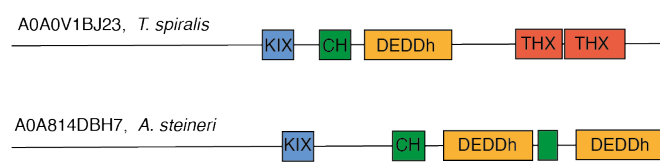

B

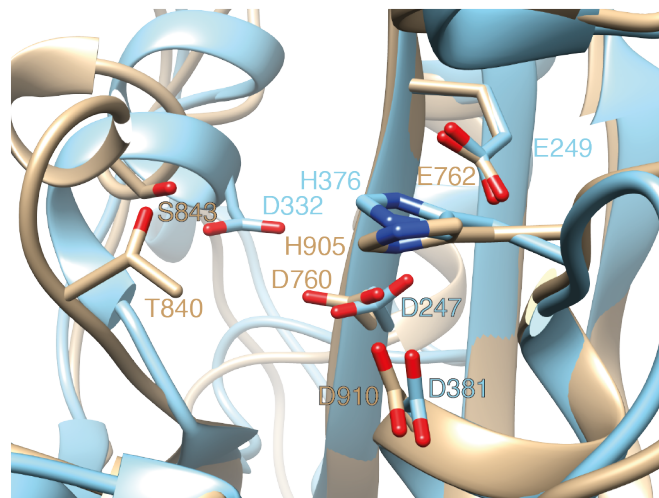

Supplement: S8 Figure — (A) Unusual domain organisations in Rex3-related proteins. The relative positions of KIX, CHORD (CH), DEDDh and thioredoxin (THX) domains in the proteins from T. spiralis and A. steineri are indicated. (B) Structural overlay of the catalytic centres of the AF models for yeast Rex3 (blue) and the A0A7J7IZG1 protein from B. neritina (brown). The positions of active site residues are shown. (PDF) [file pone.0321120.s008.pdf]
