# Supplementary material for: Conserved domains and structural motifs that differentiate closely related Rex1 and Rex3 DEDDh exoribonucleases are required for their function in yeast
Source: PLoS One. 2025 Jun 2;20(6):e0321120. doi: 10.1371/journal.pone.0321120 (PMC12129344; doi:10.1371/journal.pone.0321120)
Supplement: S1 raw images — All initial western blot and northern blot data used in this publication. (PDF) [file pone.0321120.s010.pdf]

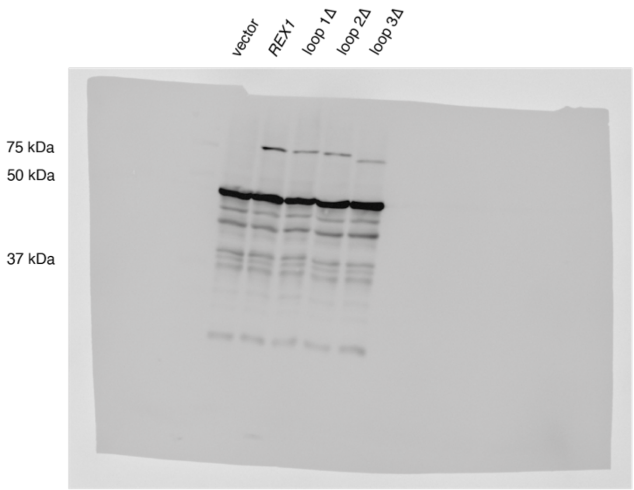

composite gel image

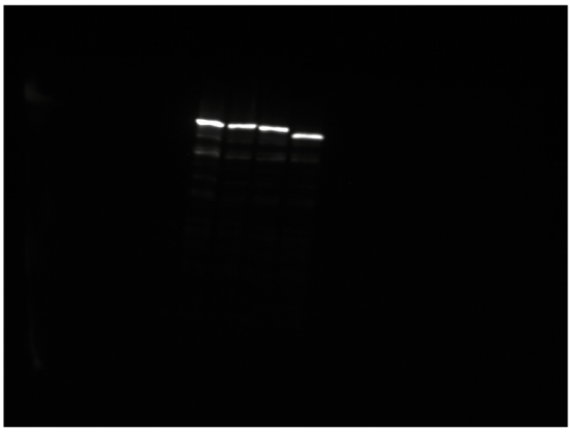

PAP antibody signal to detect Rex1 fusion proteins

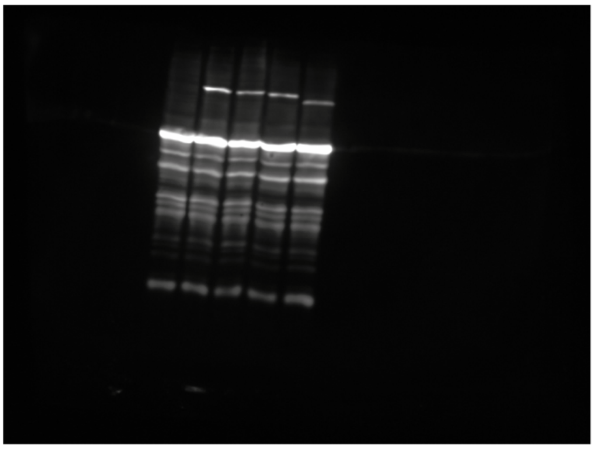

Subsequent Pgk1 antibody signal

Raw data for fig 4C. ECL western blots were imaged using an iChemi XL Gel Doc system. The original data for the PAP antibody signal and subsequent Pgk1 signal are shown, together with a composite ECL/white light image.

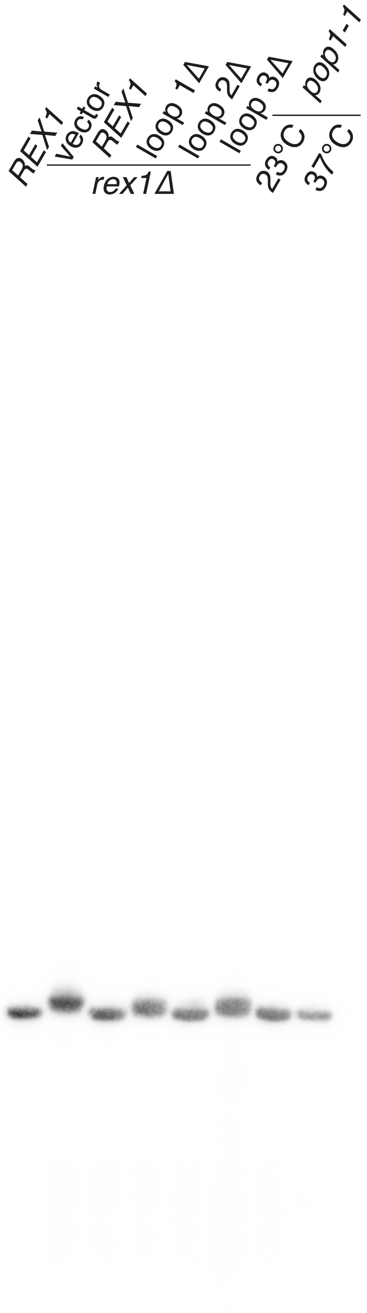

Raw data for Fig. 4D. Northern blot signal using the 5S rRNA probe. The ^{32}P signal was captured using a PhosphorImager screen and visualised using a Typhoon FLA 7000 imager.

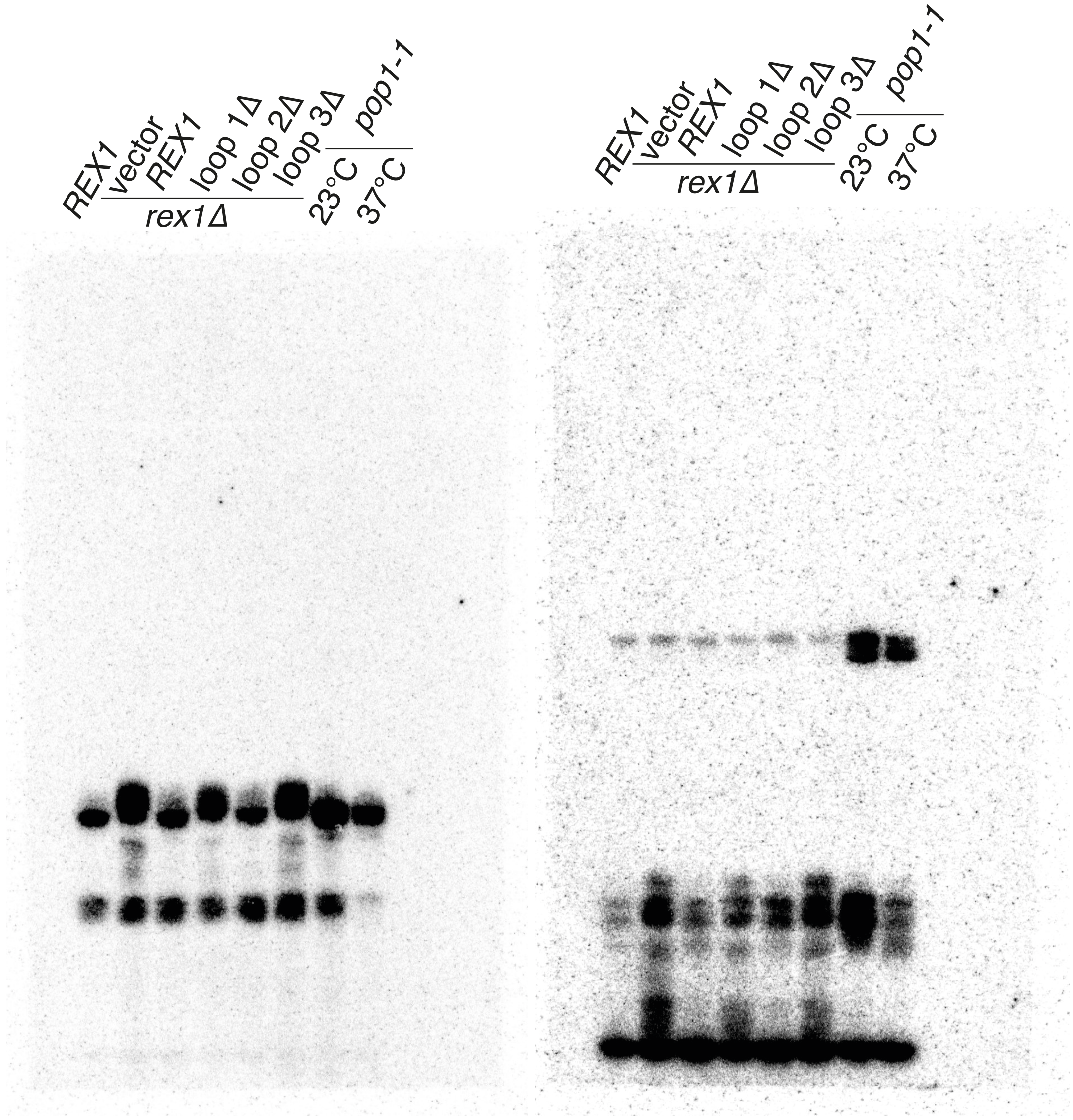

.

Raw data for Fig. 4D. Northern blot signal using the tRNA^{Arg}$_{UCU}$ probe (left-hand panel) and the tRNA^{Lys}$_{UUU}$ intron probe (right-hand panel). The ^{32}P signal was captured using a PhosphorImager screen and visualised using a Typhoon FLA 7000 imager. The blot was reiteratively hybridised with the tRNA^{Arg} probe, the tRNA^{Lys} probe and finally the 5S rRNA probe.

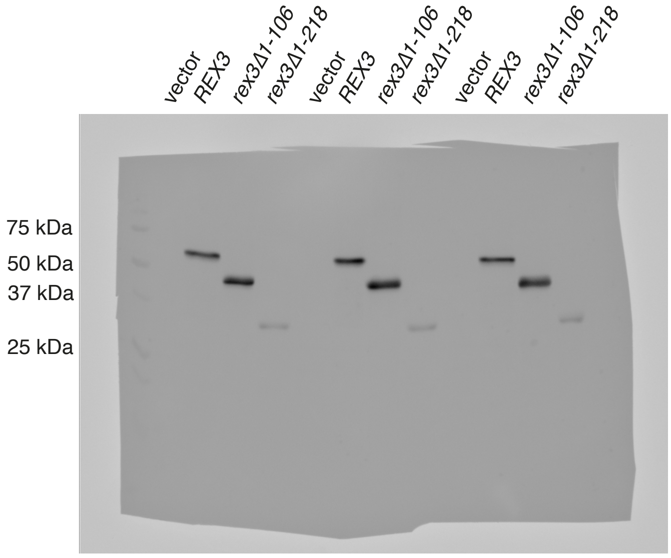

composite gel image

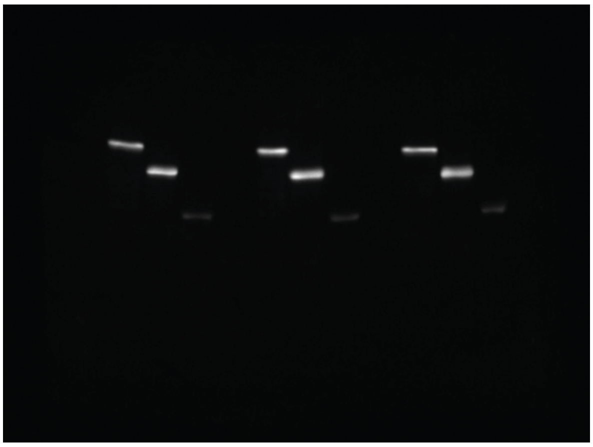

PAP antibody signal to detect Rex3 fusion proteins

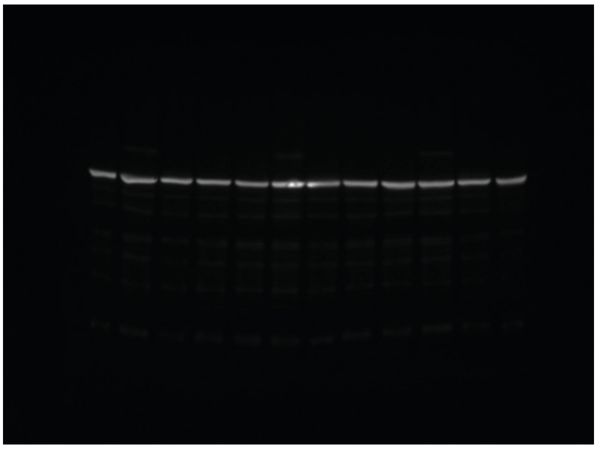

Subsequent Pgk1 antibody signal

Raw data for Fig. 6C. ECL western blots were imaged using an iChemi XL Gel Doc system. The original data for the PAP antibody signal and subsequent Pgk1 signal are shown, together with a composite ECL/white light image. The data show independent triplicate analyses used to quantify the relative amounts of Rex3 proteins. The set shown on the left-hand side were used to generate Fig. 6.

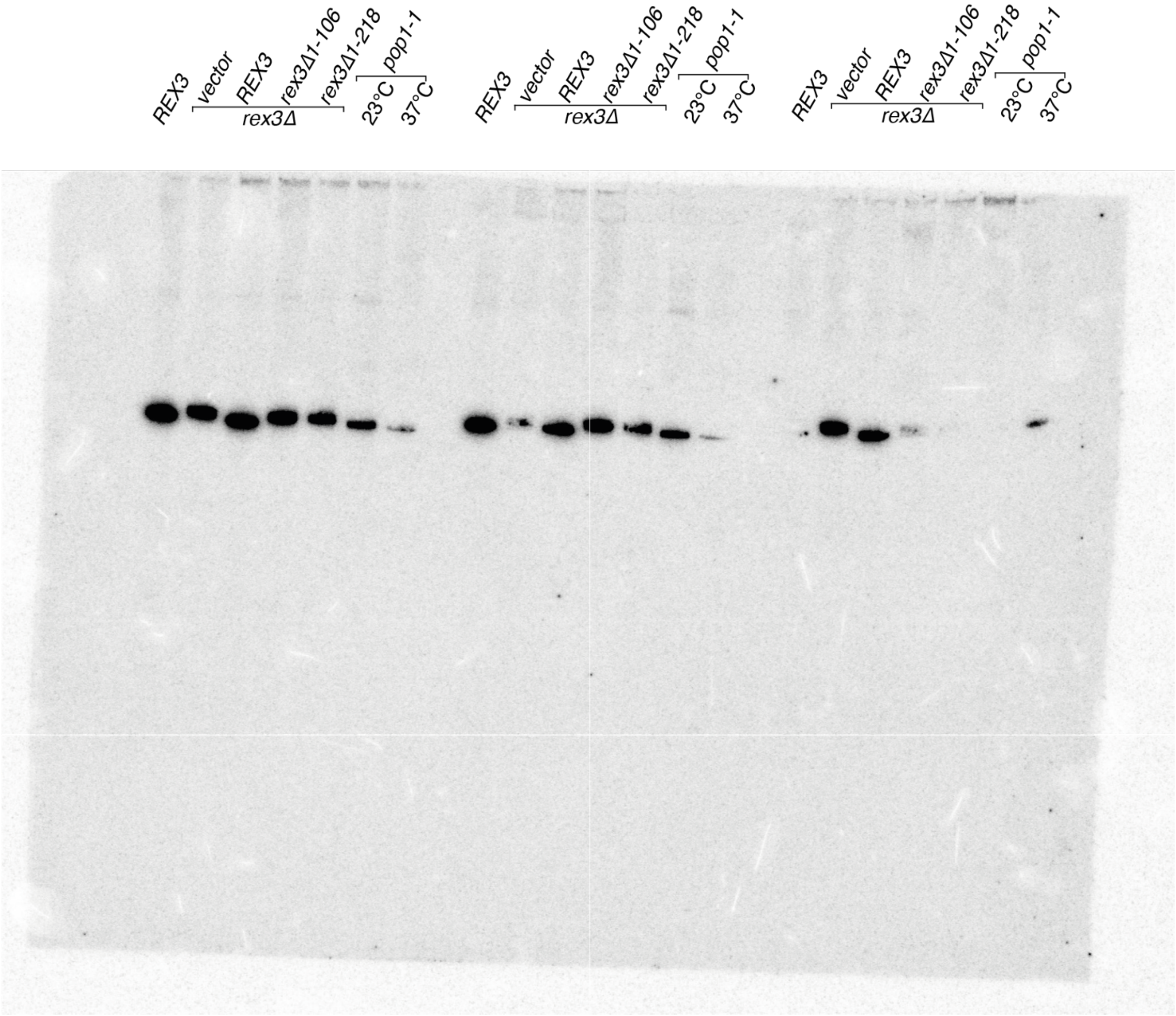

Raw data for Fig. 6B. Northern blot signal using the RNase MRP RNA probe. The ^{32}P signal was captured using a PhosphorImager screen and visualised using a Typhoon FLA 7000 imager. The same set of RNA samples was resolved in triplicate through a 6% polyacrylamide gel before transfer. The left-hand side set of samples were used to generate Fig. 6B. The same blot was then hybridised with the *SCR1* probe, shown below.

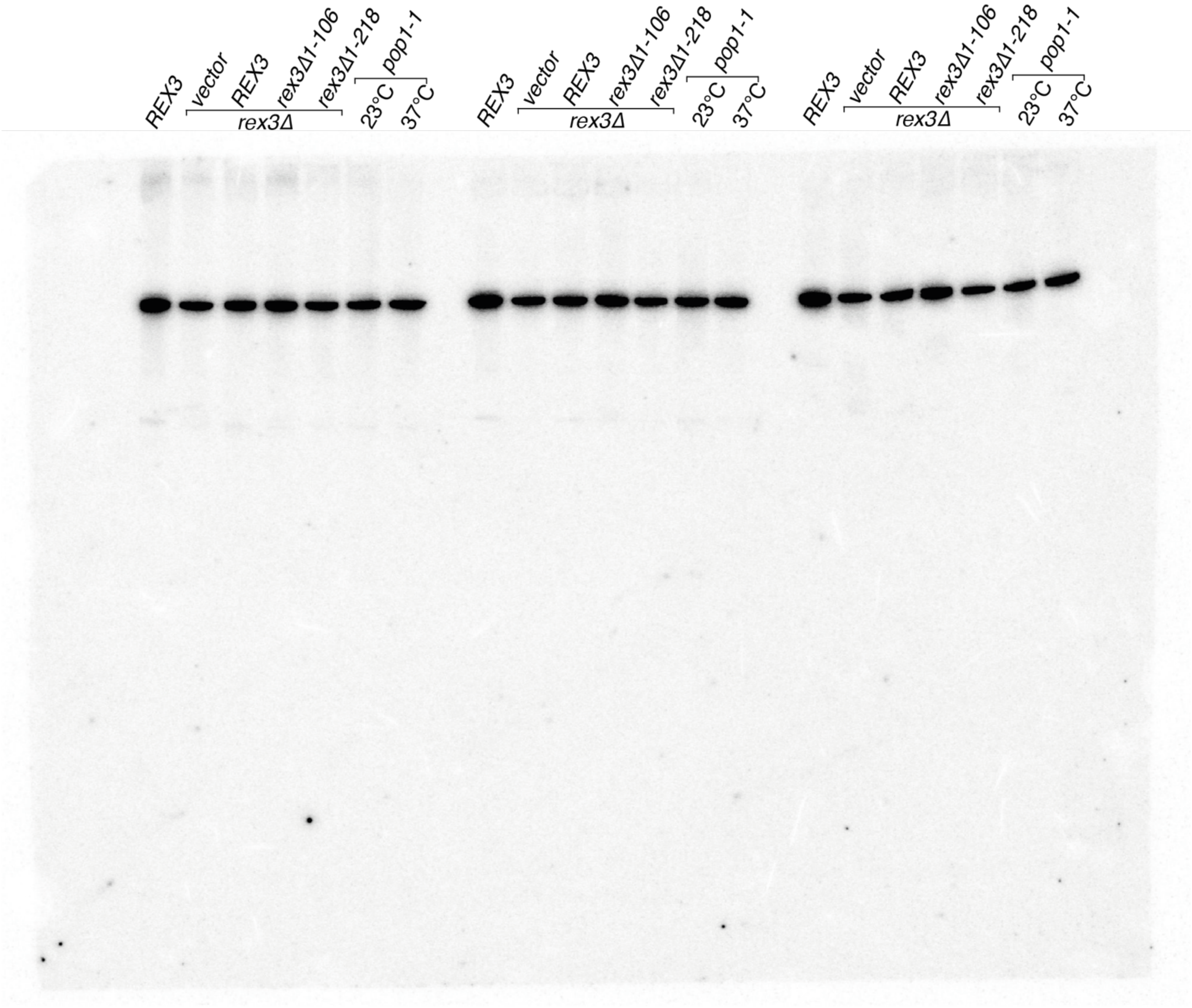

Raw data for Fig. 6B. Northern blot signal using the *SCR1* probe. Hybridisation of the blot was carried out after stripping the RNase MRP RNA signal. The ^{32}P signal was captured using a PhosphorImager screen and visualised using a Typhoon FLA 7000 imager. The left-hand side set of samples were used to generate Fig. 6B.
