# Supplementary material for: Conserved domains and structural motifs that differentiate closely related Rex1 and Rex3 DEDDh exoribonucleases are required for their function in yeast
Source: PLoS One. 2025 Jun 2;20(6):e0321120. doi: 10.1371/journal.pone.0321120 (PMC12129344; doi:10.1371/journal.pone.0321120)
Supplement: S7 Figure — Structure-based multiple sequence alignment of the KIX (upper panel) and CHORD (lower panel) domains of Rex3-related proteins. Residues are coloured by conservation. (PDF) [file pone.0321120.s007.pdf]

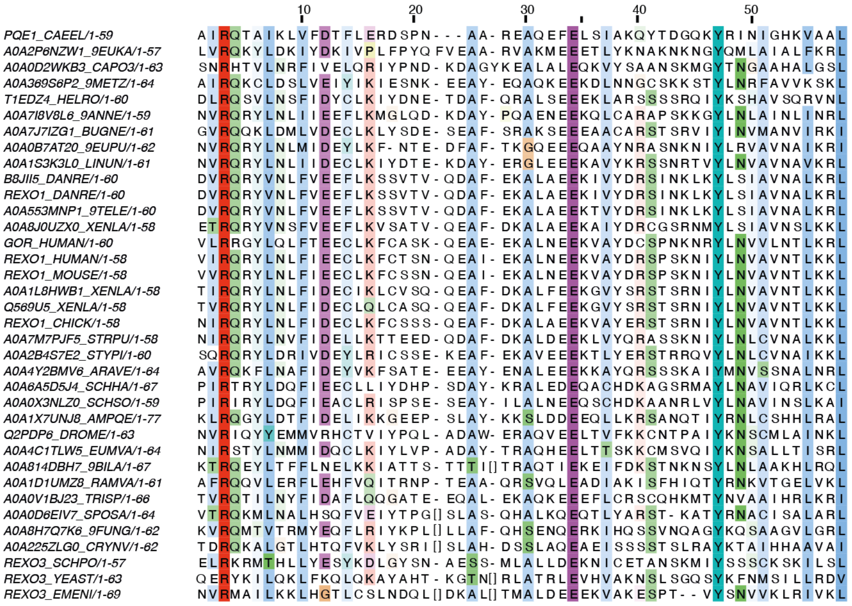
10 20 30 40 50
PQE1_CAEEL/1-59
A0A2P6NZW1_9EUKA/1-57
A0A0D2WKB3_CAPO3/1-63
A0A369S6P2_9METZ/1-64
T1EDZ4_HELRO/1-60
A0A7I8V8L6_9ANNE/1-59
A0A7J7IZG1_BUGNE/1-61
A0A0B7AT20_9EUPU/1-62
A0A1S3K3L0_LINUN/1-61
B8JII5_DANRE/1-60
REXO1_DANRE/1-60
A0A553MNP1_9TELE/1-60
A0A8J0UZX0_XENLA/1-58
GOR_HUMAN/1-60
REXO1_HUMAN/1-58
REXO1_MOUSE/1-58
A0A1L8HWB1_XENLA/1-58
Q569U5_XENLA/1-58
REXO1_CHICK/1-58
A0A7M7PJF5_STRPU/1-58
A0A2B4S7E2_STYPI/1-60
A0A4Y2BMV6_ARAVE/1-64
A0A6A5D5J4_SCHHA/1-67
A0A0X3NLZ0_SCHSO/1-59
A0A1X7UNJ8_AMPQE/1-77
Q2PDP6_DROME/1-63
A0A4C1TLW5_EUMVA/1-64
A0A814DBH7_9BILA/1-67
A0A1D1UMZ8_RAMVA/1-61
A0A0V1BJ23_TRISP/1-66
A0A0D6EIV7_SPOSA/1-64
A0A8H7Q7K6_9FUNG/1-62
A0A225ZLG0_CRYNV/1-62
REXO3_SCHPO/1-57
REXO3_YEAST/1-63
REXO3_EMENI/1-69

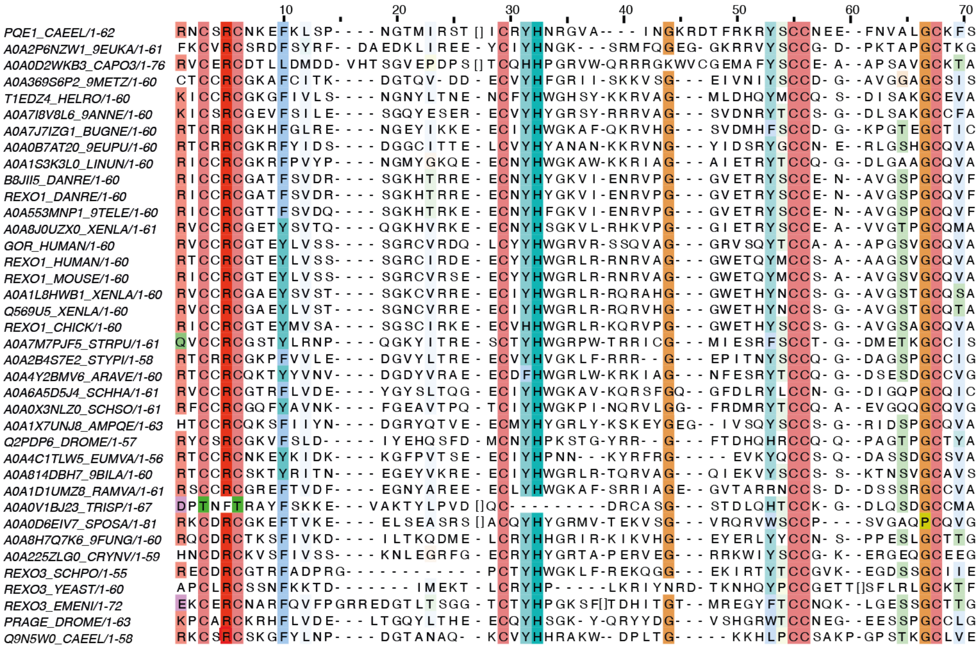
10 20 30 40 50 60 70
PQE1_CAEEL/1-62
A0A2P6NZW1_9EUKA/1-61
A0A0D2WKB3_CAPO3/1-76
A0A369S6P2_9METZ/1-60
T1EDZ4_HELRO/1-60
A0A7I8V8L6_9ANNE/1-60
A0A7J7IZG1_BUGNE/1-60
A0A0B7AT20_9EUPU/1-60
A0A1S3K3L0_LINUN/1-60
B8JII5_DANRE/1-60
REXO1_DANRE/1-60
A0A553MNP1_9TELE/1-60
A0A8J0UZX0_XENLA/1-61
GOR_HUMAN/1-60
REXO1_HUMAN/1-60
REXO1_MOUSE/1-60
A0A1L8HWB1_XENLA/1-60
Q569U5_XENLA/1-60
REXO1_CHICK/1-60
A0A7M7PJF5_STRPU/1-61
A0A2B4S7E2_STYPI/1-58
A0A4Y2BMV6_ARAVE/1-60
A0A6A5D5J4_SCHHA/1-61
A0A0X3NLZ0_SCHSO/1-61
A0A1X7UNJ8_AMPQE/1-63
Q2PDP6_DROME/1-57
A0A4C1TLW5_EUMVA/1-56
A0A814DBH7_9BILA/1-60
A0A1D1UMZ8_RAMVA/1-61
A0A0V1BJ23_TRISP/1-67
A0A0D6EIV7_SPOSA/1-81
A0A8H7Q7K6_9FUNG/1-60
A0A225ZLG0_CRYNV/1-59
REXO3_SCHPO/1-55
REXO3_YEAST/1-60
REXO3_EMENI/1-72
PRAGE_DROME/1-63
Q9N5W0_CAEEL/1-58
