# Supplementary material for: Conserved domains and structural motifs that differentiate closely related Rex1 and Rex3 DEDDh exoribonucleases are required for their function in yeast
Source: PLoS One. 2025 Jun 2;20(6):e0321120. doi: 10.1371/journal.pone.0321120 (PMC12129344; doi:10.1371/journal.pone.0321120)
Supplement: Attachment — Submitted filename: response to reviewers.docx [file pone.0321120.s012.docx]

Dear Editor

I am submitting our revised version of the manuscript PONE-D-24-32419 entitled "Conserved domains and structural motifs that differentiate closely related Rex1 and Rex3 DEDDh exoribonucleases are required for their function in yeast". The manuscript has been extensively revised in response to the comments of the reviewers, together with the editor's comments.

Response to Reviewer 1

*The manuscript by Daniels, et al., entitled “Conserved domains and structural motifs that differentiate closely related Rex1 and Rex3 DEDDh exoribonucleases are required for the function in yeast” describes phylogenetic and structural analysis of the DEDD family of exonucleases. The authors identify two distinct clades within a subgroup of the DEDD family. They further use mutagenesis approaches to demonstrate that regions outside the DEDD domains play critical roles in in vivo RNA processing events.

The overall conclusions, including the classification of clades with distinct structural features, appears to be well supported. The study provides a useful description of Rex1 and Rex3 exoribonucleases. However, I have concerns with the presentation of the structural data, as described below, which should be addressed before acceptance.

The structural analysis of relies heavily on AlphaFold generated models. The authors should clearly indicate what version of AlphaFold they are using. There are several descriptions of AlphaFold models/structural clusters that aren’t referenced in a figure or supplemental data (for example: pg 7, ln 145; pg 9, ln 214; pg 13, ln 319). Can the predicted models/coordinates be provided as supplemental data and summarized in a figure? Perhaps the final panel in Fig 4A starts to address the question, but it isn’t referenced in relevant places in the main text and the source data is still missing. (Also, please clearly define the confidence thresholds used for the coloring scheme in Fig 4A.) Some AlphaFold models are presented in figures, colored by confidence scores. At other times, the average pLDDT scores for the entire protein are reported (see pg 12, ln 290). I understand the difficulty of succinctly presenting all the data, but there should be an effort to be more consistent. This further underscores the need to make the predicted structures available (with confidence scores).

Figure 1 is extremely difficulty to follow. Panel A: Are all 3 structures AlphaFold models? It seems strange to have different coloring schemes for the structures. The position of the DEDDh with respect to the AlkP fold is difficult to discern. It might help to present the entire structure first, possibly color coded by domain. It would also be helpful to use the same color scheme in the topology diagram. Panel B: Is there a reason the AlkP domain isn’t included in the domain structure figure? What is the relationship between the structures in Panel B and Panel C? (I think the view has simply been rotated and the DEDD removed. Some kind of visual cue would be helpful.) Overall, the idea of a common AlkP fold with a DEDDh insertion is very challenging to understand as presented.*

We thank the reviewer for their comments.

The AlphaFold models used were retrieved from the version 4 database. This is now explicitly stated on line 96 and 689.

We have extensively revised the manuscript to include source data that supports structure similarities, as requested by the reviewer. In some cases this is within the expanded set of data shown in the Figures but largely within supplemental Tables and Figures. AlphaFold database accession codes or PDB chain identifiers are included in the Tables. The supplementary Tables include structure comparison Foldseek and MMseqs2 hits for Rex1, Rexo5 and Rex3 (S1 Table), AlphaFold database hits for iPGM (S1 Table), PUF60 RRMs (S3 Table), and the KIX and CHORD domains (S4 Table), as well as PDB hits for Rexo5 RRMs (S3 Table), and the KIX and CHORD domains (S4 Table). S6 Table includes an important structure summary of the AlkP and RRM domains within Rex1 proteins and the KIX and CHORD domains within Rex3 proteins that provides AlphaFold database IDs and InterPro database identifiers, defines the residues within each structural element and provides individual pLDDT scores. The revised figures now include a comparison of AlphaFold models of yeast Rex1 homologues (Fig 3A), the CHORD domains of Rex3, REXO1 and RAR1 (Fig 5C) and AlphaFold models of the AlkP domain of Rex1 homologues in trypanosomes (S2 Figure).

The confidence scores of the AlkP, RRM, KIX and CHORD domains indicated in Fig 4A of the original manuscript (now Fig 7A) are indicated in the figure legend. Moreover, we have included pLDDT scores for each domain. These are also included in the S6 Table, which summarises the domains for each Rex1 and Rex3 homologue analysed.

Within the text, the confidence of a given structure is supported by giving the pLDDT score for either a specific defined number of residues or the full length of the protein. We have provided such confidence cores using a consistent style throughout the revised manuscript.

Structure comparisons of AlphaFold models are presented in Fig 1 and Fig 3 in the revised manuscript in a consistent manner, whereby the models are colour-coded according to the protein domain, as suggested by the reviewer; the DEDDh domain is colured green, the N- and C-terminal regions of the AlkP domain are coloured blue and red, respectively, and the RRMs are coloured gold. The models in the revised Figure 1 are all in the same colour scheme. Structures for iPGM and AlkP are from crystallographic models. This is clearly explained in the figure legend of the revised manuscript and referred to in the text. We have provided an initial view of the complete structure of the protein (Fig 1A), as suggested by the reviewer. The revised colour scheme emphasises the fact that the DEDDh domain is "inserted" into the AlkP domain. We have included the AlkP domain in the domain structure schematic for Rex1-related proteins (Fig 1D), as suggested by the reviewer. We present models of the complete human and fly Rexo5 proteins (lacking the unstructured N-terminal regions) for the reader to be able to compare them with the structure of yeast Rex1. We also show structures of the AlkP domain of the fly Rexo5 protein and the equivalent portion of the human protein that are viewed from the same perspective as that shown for the yeast protein. This is to enable a comparison of the fold of the AlkP domain, which is not clear in the images of the whole protein. We have included PAE maps of the proteins in the revised Fig 1. These maps portray well the interactions between the N- and C-terminal regions of the protein and emphasise that the domain packing within the proteins vary. This point is important, as the precise alignment of the AlkP and DEDDh domains has varying degrees of confidence in the models of the yeast, fly and human proteins, which is explicitly noted in the revised manuscript.

We sincerely thank the reviewer for their comments, which have helped us to significantly improve the manuscript.

Response to Reviewer 2

*The manuscript by Daniels et al. “Conserved domains and structural motifs that differentiate closely related Rex1 and Rex3 DEDD exoribonucleases are required for their function in yeast” addresses the characteristic structural features of Rex1 and Rex3 yeast ribonucleases. The authors employed the Alphafold to predict the high confidence domain structure of the representative proteins from the subgroup with catalytic DEDD domain including the yeast enzymes Rex1 and Rex3, the metazoan REXO1 and Rexo5 proteins, and the plant protein Sdn5. Comparison of protein structure models revealed that this group can be differentiated into two distinct clades - Rex1/Rexo5/Sdn5 and Rex3/REXO. Structural modeling of Rex1 revealed three surface loops directed towards the DEDD domain, one of which forms an extended helical arch that is found in homologues across fungi and plants. Molecular analysis showed that the arch and an adjacent loop are required for Rex1-mediated processing of 5S rRNA and tRNA. Rex3-related proteins contain a KIX domain interacting with CREB kinase-inducible domain and a cysteine- and histidine-rich domain (CHORD) adjacent to a C-terminal DEDD domain. Deletion of the N-terminal region spanning the KIX domain blocked Rex3 function in RNase MRP processing.
Altogether, this work identifies evolutionarily conserved structural hallmarks within Rex1 and Rex3 proteins and demonstrates that specific features are required for Rex1- and Rex3-mediated RNA processing pathways in vivo.

The results are interesting and but publication in PLOS ONE requires some additional work, as specified below.
Critical comments:
1. Figure 2D shows complementation of the synthetic lethal phenotype of double mutant rex1 delta rrp47 delta by rex1 deletion mutants. This experiment lacks a negative control. This is because the plasmid shuffle assay is not a proper approach for complementation study. I would recommend tetrad analysis of the meiotic progeny of heterologous diploid rex1 delta rrp47delta/rex1 delta RRP47 transformed with empty vector and plasmids with REX1 or rex1 deletions.
2. Figure 2C presents Northern blot showing that the molecular phenotypes of deletion mutants varied; loop 2 –delta is like wild type, whereas loop 1 and loop 3 delta are like rex1delta. However, as stated in the abstract only one loop, adjacent to the helical arch, is required for Rex1-mediated 5S rRNA and tRNA processing. Please explain this inconsistence.
3. Why the zzRex1 protein migrates as a lower band in loop3 delta mutant as shown on Figure 2C?
4. Which tag was used for epitope-tagging of Rex1 and Rex3? What “zz” means?
5. Figure 2E: The experiment lacks a negative control What effects of rex3 mutants on SNR8 and SNR30 were expected? Why the level of delta1-218 mutated protein is so low? It desires some comment.
6. How the authors understand the statement “stable RNA processing” which they use several times?
7. The description of the domain structure of Rex3 and presentation on Figure 3A are unclear to me. Text is too long and many short-cuts were used, the idea is lost.*

We thank the reviewer for their comments.

1. We have included the plasmid shuffle assay data (revised fig 4A) in the revised manuscript. This clearly shows that there is no detectable growth of the plasmid shuffle strain on medium containing FOA when it is transformed with the vector control. As this point was also raised by the editor, we nevertheless carried out meiotic progeny analyses of a heterozygous diploid strain. We were limited to carrying out a random spore analysis rather than tetrad analysis due to the germination efficiency of the available heterozygous diploid strain. We analysed over 80 randomly selected progeny from transformants of the diploid strain harbouring the wild-type Rex1, each loop mutant, the vector control and an untransformed control. The data (Fig 4B, Table 1) agree with published data (Peng et al., 2003) that *rex1∆ rrp47∆* double mutants are inviable and support the conclusion that each loop mutant analysed complements this growth phenotype.

2. The abstract states that three surface loops are directed towards the DEDD domain, one of which forms an arch. The abstract then states that the arch and an adjacent loop are required for RNA processing. The text is consistent - the misunderstanding stems from the fact that the arch is one of the loops. We have altered the text slightly to emphasise this. The abstract now reads "The AlkP domain ... contains three surface loops..., one of which forms an extended helical arch... We show that *this* arch and an adjacent loop are required for Rex1-mediated processing..."

3. The loop 3∆ mutant has the largest deletion, such that the protein is 3-4 kDa less than the wild-type protein and the other mutants and thus migrates faster upon SDS-PAGE analysis. We have made this point explicitly in the revised manuscript (line 336).

4. The zz tag contains two copies of the z domain of Protein A from *Staphylococcus aureus*. This is explicitly stated in the revised manuscript (line 708).

5. The data in Fig 3E of the original manuscript (Fig 6B of the revised manuscript) shows the expression of RNase MRP RNA in the *rex3∆* mutant and in strains expressing either wild-type Rex3 or the deletion mutants. The observation is that the RNase MRP RNA is longer in the *rex3∆* mutant and cells expressing the Rex3 mutants than in cells expressing the wild-type Rex3 protein. It is not clear what is meant by an additional negative control, as the *rex3∆* mutant is the sample that does not express any Rex3 protein. We have included samples from the *pop1-1* temperature-sensitive mutant as a control for the specificity of the northern hybridisation. As predicted, RNase MRP RNA is depleted in the *pop1-1* mutant during growth at the nonpermissive temperature. It is not feasible to analyse an RNA from a strain that does not express RNase MRP RNA, as the *NME1* gene that encodes RNase MRP RNA is essential for viability.

We have described more clearly in the revised manuscript (p 22) our reasoning for analysing the expression levels of the snoRNAs snR30 and snR8. We present data that Rex3 contains a CHORD domain. These domains form protein interactions with CS domains. The yeast protein Shq1 contains a CS domain and is required for assembly of snoRNAs into RNA/protein complexes. We wanted to test the hypothesis that Rex3 might interact via its CHORD domain with Shq1 and thereby couple RNA processing with assembly of the processed RNA into RNP complexes. There is no clear effect on snR30 or snR8 levels in the rex3∆ mutant but we feel that this negative data is nevertheless worthy reporting.

There are two potential reasons why the level of the *rex3* ∆1-218 mutant is markedly reduced. One is that residues 119-142, which are modelled to be packed against the DEDDh domain, are required for its stable expression. The other is that the CHORD domain itself is required for stable protein expression. Characterised CHORD domain proteins function as part of Hsp90 protein chaperone complexes. These explanations are discussed in greater detail in the revised manuscript (p 22).

6. The phrase "stable RNA processing" relates to post-transcriptional RNA processing events that occur on stably expressed RNAs. We have rephrased this in the revised manuscript to be more explicit, referring for example to "the processing of noncoding transcripts to generate functional, stably expressed RNAs" (line 59).

7. The description of Fig 3A (Fig 5A in the revised manuscript) describes the presence of the KIX domain and CHORD domain in Rex3. It outlines homology with characterised proteins containing these domains and describes conservation both in terms of protein structure and sequence homology. The text then discusses the potential functional importance of the KIX domain to Rex3 function in coupling RNA processing to transcription. Finally, the section describes the conservation of KIX and CHORD domains in both Rex3- and REXO1-related proteins. We feel that these are important aspects that need to be covered in the manuscript.

Editors comments

1. *In my independent review, I was not convinced by the Northern analysis that the authors conclude are indicative of processed and unprocessed tRNA forms. The combination of a lack of resolution and a comparison with predicted mobilities rather than a side by side comparison with precursor and product forms result is inadequate.*

2. *I fully concur with Reviewer 2’s issue on the interpretation of the complementation results in Figure 2D and Figure 3E.  A negative control is required to show that actual complementation is taking place. The heterozygous analysis of meiotic progeny would be the best approach.*

3. *To eliminate gene expression effects of plasmids, it would also be best to integrate the tagged deletion constructs that appear to be used rather than use plasmids. While the plasmid shuffles are highly suggestive, they are not ideal and require assumptions based on published data.*

4. *I think that the use of the RRP4 promoter as opposed to using the endogenous promoter is problematic, (although used by the author in published studies) Are these at higher/lower abundance than the endogenous protein> This is essential information to verify the appropriateness of RRP4 promoter use.*

5. *As pointed out by Review 1, the AlphaFold data is difficulty to interpret without appropriate referencing, statistical analysis, and a lack of consistency in the presentation of the data.*

We thank the editor for their comments.

1. It was not our intention to provide a detailed analysis of tRNA processing , as this has already been extensively studied and reported by a number of independent research groups. Our main conclusion that the *rex1* loop1∆ and loop 3∆ mutants do not support 5S rRNA and tRNA processing whereas the loop 2∆ mutant does is based on a simple comparison of the observed northern blot hybridisation patterns with that of the *rex1∆* mutant and strains expressing wild-type Rex1. However, we did feel that we should describe the main RNAs observed. To address the editor's comments, we have extended the analyses. The northern blot analyses shown in Fig 4 of the revised manuscript include a sample from a wild-type strain and a *pop1-1* strain that is defective in RNase P activity. The *pop1-1* mutant accumulates the primary transcript and the processing intermediate (substrates of RNase P) when exhibiting a nonconditional defect at the permissive temperature, and is depleted of the processing intermediate (also an RNase P product) at the nonpermissive temperature. We also hybridised the blot with probes for 5.8S rRNA (158 nt) and U24 snoRNA (89 nt), which provide reasonable size markers for the dicistronic tRNA transcript (160 nt) and the tRNA^Arg^_UCU_ processing intermediate (~ 90 nt), while 5S (121 nt) provides a good size marker for the tRNA^Lys^_UUU_ primary transcript (~ 120 nt)

2. This point has been addressed above.

3/4. It is true that expression from the homologous chromosomal locus would be more likely to give physiologically appropriate expression levels than from a plasmid-borne gene. The expression of Rex1 from the plasmid is markedly higher than what is observed from the chromosomal homologous promoter, as stated in the text (line 340). However, our main conclusions are that the rex1 loop 1∆ and loop 3∆ mutants and the rex3 ∆1-106 mutant have loss of function phenotypes. It is not clear how an increased expression of the mutant protein would be responsible for a loss of function phenotype, particularly when the expression of the mutant protein is comparable to that of the wild-type protein under the control of the same expression system that does not exhibit the defect.

We have performed meiotic segregant analyses, as requested. These data support the published synthetic lethality of the *rex1∆ rrp47∆* double mutant and demonstrate that the rex1 loop mutants complement this phenotype.

5. These points have been addressed above. All the source data for AlphaFold database and PDB database searches have been included as supplementary Tables or in the Figures and the panels in the revised Figure 1 allow for a much clearer comparison of the protein structures.
