# Supplementary material for: Conserved domains and structural motifs that differentiate closely related Rex1 and Rex3 DEDDh exoribonucleases are required for their function in yeast
Source: PLoS One. 2025 Jun 2;20(6):e0321120. doi: 10.1371/journal.pone.0321120 (PMC12129344; doi:10.1371/journal.pone.0321120)
Supplement: Attachment — Submitted filename: response_to_reviewers_auresp_2.docx [file pone.0321120.s013.docx]

Dear Editor

I am submitting our revised version of the manuscript PONE-D-24-32419 entitled "Conserved domains and structural motifs that differentiate closely related Rex1 and Rex3 DEDDh exoribonucleases are required for their function in yeast". We very much appreciate the rapid processing of the submitted revised manuscript.

Reviewer 1 commented that all the issues had been addressed and we thank this reviewer for their constructive comments and input in the review process. We have addressed the comment raised by yourself, and previously by reviewer 2, regarding the absence of a wild-type sample in the northern blot analysis of the rex3 mutant phenotype. We have now rectified this omission and trust that the revised manuscript fully addresses the issue raised.
